# The Molecular Mechanisms Underlying Iron Deficiency Responses in Rice

**DOI:** 10.3390/ijms21010043

**Published:** 2019-12-19

**Authors:** Qian Li, Lei Chen, An Yang

**Affiliations:** 1 State Key Laboratory of Vegetation and Environmental Change, Institute of Botany, the Chinese Academy of Sciences, Beijing 100093, China; liqian18@nwafu.edu.cn; 2 Guangdong Provincial Key Laboratory of Biotechnology for Plant Development, School of Life Sciences, South China Normal University, Guangzhou 510631, China; chenlei@frontier-ag.com

**Keywords:** rice (*Oryza sativa*), Fe deficiency, strategy II, Fe acquisition, transporters, transcription factors, phytohormones

## Abstract

Iron (Fe) is an essential element required for plant growth and development. Under Fe-deficientconditions, plants have developed two distinct strategies (designated as strategy I and II) to acquire Fe from soil. As a graminaceous species, rice is not a typical strategy II plant, as it not only synthesizes DMA (2′-deoxymugineic acid) in roots to chelate Fe^3+^ but also acquires Fe^2+^ through transporters *OsIRT1* and *OsIRT2*. During the synthesis of DMA in rice, there are three sequential enzymatic reactions catalyzed by enzymes NAS (nicotianamine synthase), NAAT (nicotianamine aminotransferase), and DMAS (deoxymugineic acid synthase). Many transporters required for Fe uptake from the rhizosphere and internal translocation have also been identified in rice. In addition, the signaling networks composed of various transcription factors (such as IDEF1, IDEF2, and members of the bHLH (basic helix-loop-helix) family), phytohormones, and signaling molecules are demonstrated to regulate Fe uptake and translocation. This knowledge greatly contributes to our understanding of the molecular mechanisms underlying iron deficiency responses in rice.

## 1. Introduction

Iron (Fe) is one of the key micronutrients required for various metabolic processes in plants [1,2]. For example, chlorophyll synthesis will be retarded when exposed to an Fe deficiency, leading to interveinal chlorosis in plant leaves and reduced crop yields [1]. Like in plants, Fe is also of great importance for human health. Bioavailable Fe deficiency in food will lead to anemia, one of the top ten health problems in humans at present [3]. It is estimated that at least 2 billion people around the world are affected by Fe-associated anemia, with women of child-bearing age being particularly affected [1,3]. Therefore, the biofortification of Fe content in crops will have huge benefits for human health around the world.

Although Fe is the second most abundant metal element in the earth’s crust next to aluminum, it mostly exists in the form of insoluble hydroxides and oxides, especially in neutral-to-alkaline soils, which are not bioavailable for plants [4,5]. To cope with low Fe bioavailability in soil, all non-graminaceous monocots and dicots have developed a reduction strategy (named strategy I), while graminaceous plants, including most grain crops, have developed a chelation strategy (named strategy II) [6,7]. In strategy I, H^+^-ATPases-mediated protons are extruded from the root cell, which leads to the acidification of the rhizosphere and subsequently prompts Fe solubilization [8,9]. Then, the ferric iron (Fe^3+^) is reduced to ferrous iron (Fe^2+^) by means of a plasma membrane-located ferric chelate enzyme FRO (ferric reductase oxidase) [10]. In strategy I plants, this FRO-catalyzed reduction of Fe^3+^ to Fe^2+^ is the rate-limiting step for Fe uptake [10]. Lastly, Fe^2+^ is transported across the plasma membrane by iron-regulated transporter 1 (IRT1) [11,12]. In strategy II, plants synthesize and secrete mugineic acid (MA) family phytosiderophores in the root to chelate Fe^3+^ [13,14]. Although various MAs exist among different graminaceous species, all MAs are synthesized from S-adenosyl-L-methionine [15,16,17].

Rice, a graminaceous species, is an important staple crop and feeds more than half of the world’s population. However, rice is not a typical strategy II plant. Under conditions of low Fe availability, rice plants can only synthesize DMA (2′-deoxymugineic acid) in roots to chelate Fe^3+^ in the rhizosphere [13,14]. In addition, rice is equipped with strategy I to acquire Fe^2+^, which is mediated by two Fe^2+^ transporters, *OsIRT1* and *OsIRT2*, in root cells [18]. This is conceivable because rice and its wild relatives need to adapt to waterlogged wetlands, where most iron elements exist as Fe^2+^ due to the low redox potential [18,19]. Although rice plants possess dual Fe acquisition strategies, they are highly susceptible to low Fe conditions in calcareous soils [20,21]. Furthermore, the Fe concentration in polished rice seeds is very low, which cannot satisfy the micronutrient requirements of a human diet [22]. More seriously, the concentration of Fe in rice grains is decreasing by the enriched atmospheric CO_2_ due to anthropological activities, which will aggravate micronutrient deficiencies in human nutrition in rice-dependent countries [23,24,25]. To address these problems, we need to biofortify Fe concentrations in rice grains by means of genetic breeding, which relies on determining the mechanisms underlying Fe deficiency responses in rice. Fortunately, a number of genes involved in iron uptake, transport, and regulation have been identified in rice (Table 1). In this review, we summarize the recent progress involved in the Fe signaling networks and homeostasis in rice, and particularly, the detailed knowledge of regulatory roles of phytohormones and messenger molecules in Fe homeostasis.

## 2. Molecular Processes During Iron Acquisition from the Rhizosphere

**Figure 1:**
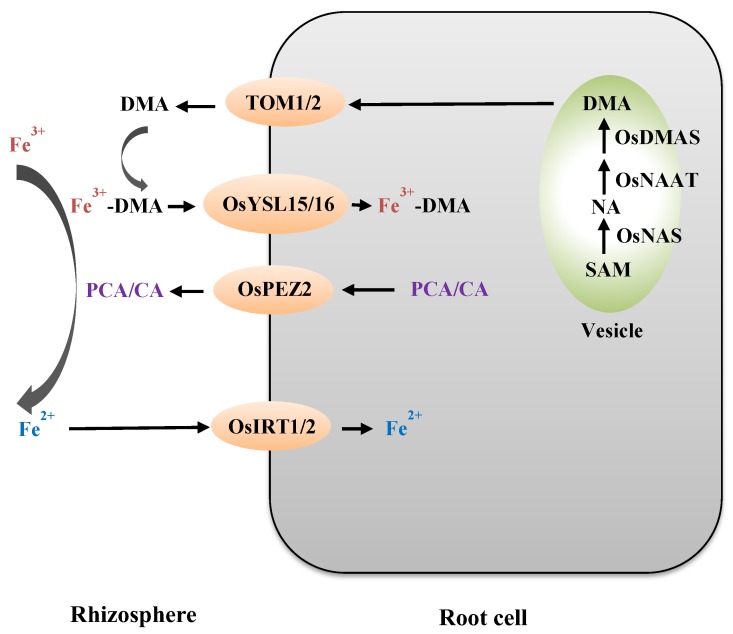
A simplified model of Fe uptake from the rhizosphere in rice. Rice not only synthesizes DMA in roots to chelate Fe^3+^ but also acquires Fe^2+^ through transporters *OsIRT1* and *OsIRT2*. During the synthesis of DMA, there are three sequential enzymatic reactions catalyzed by the enzyme, OsNAS, OsNAAT, and OsDMAS. The TOM (transporter of mugineic acid family phytosiderophores) and OsYSL transporters are required for Fe uptake from the rhizosphere. OsPEZ2s are phenolic efflux transporters responsible for the transport of protocatechuic acid/caffeic acid (PCA/CA). The root cell is shown in the grey rounded rectangle. The vesicle is shown in the light green ellipse. Transporters are shown in the light orange ellipses.

In strategy II plants, MA family phytosiderophores are synthesized in vesicles and secreted in the root to chelate Fe^3+^ [13,14] (Figure 1). Different species and genotypes within a species can differ in the types of MAs secreted in their roots [57]. For example, rice, corn, and wheat secret 2′-Deoxymugineic acid (DMA) to chelate Fe^3+^, while barley secrets other types of MAs, including MA, 3-hydroxymugineic acid, and 3-epi-hydrdeoxymugineic acid, to chelate Fe^3+^ [57]. During the synthesis of DMA in rice, three sequential enzymatic reactions are catalyzed by nicotianamine (NA) synthase (NAS), NA aminotransferase (NAAT), and deoxymugineic acid synthase (DMAS) [26,27,28]. NAS, encoded by three genes (Os*NAS1*/*NAS2*/*NAS3*), is the key enzyme during the synthesis of DMA in rice [26]. Under Fe deficiency conditions, the expression level of genes encoding NAS, NAAT, and DMAS is increased [58,59]. After being synthesized in root cells, DMAs are secreted into the rhizosphere via transporter TOMs (transporter of mugineic acid family phytosiderophores) (Figure 1). The expression levels of *TOM1* and *TOM2* are induced by Fe-deficient treatment. Alteration to the expression levels of *TOM1* or *TOM2* in transgenic rice could change the tolerance to Fe deficiency [29,30]. Then, Fe^3+^-DMAs are formed in the rhizosphere and transported into the root cells by YSL (yellow stripe1-like) proteins [60]. YSL proteins are homologous proteins of YS1 (yellow stripe1) in maize. YS1 was first identified in a maize *ys1* mutant, which exhibited Fe chlorosis due to the impairment of Fe^3+^-phytosiderophore uptake [61]. In addition to Fe^3+^-phytosiderophore uptake, the YSL protein is also involved in Fe^2+^-NA, Cu^2+^-NA, and Mn^2+^-NA translocation. In rice, there are 18 genes encoding the YSL transporter. Among these transporters, OsYSL15 and OsYSL16 are involved in Fe^3+^-DMA uptake in rice [35,36]. Furthermore, rice can extrude protocatechuic acid (PCA) and caffeic acid (CA) to enhance Fe solubility through the phenolic efflux transporter, OsPEZ2 [40,62] (Figure 1).

Despite being a strategy II plant, rice also absorbs Fe^2+^ directly via the OsIRTl transporter [18,63] (Figure 1). Moreover, there are two FRO2-like genes, *OsFRO1* and *OsFRO2*, in the rice genome [18]. These two genes were first identified by Ishimaru et al. and possibly have no reductase activities in the rice root [18]. Recently, Li et al. observed that OsFRO1 is localized in the vacuolar membrane in rice protoplasts [64]. They found that OsFRO1 has all the necessary motifs to act as FRO enzyme. In contrast, no complete transmembrane domain exists at the N-terminal of OsFRO2 protein. The concentrations of Fe in the overexpressed *OsFRO1* and RNAi lines are higher and lower than that in the wild-type plants under Fe excess conditions, respectively. These results suggest that OsFRO1 could mediate the reduction of Fe^3+^ to Fe^2+^ in the vacuole, thus regulating Fe homeostasis in cells [64].

## 3. Internal Iron (Fe) Translocation

**Figure 2:**
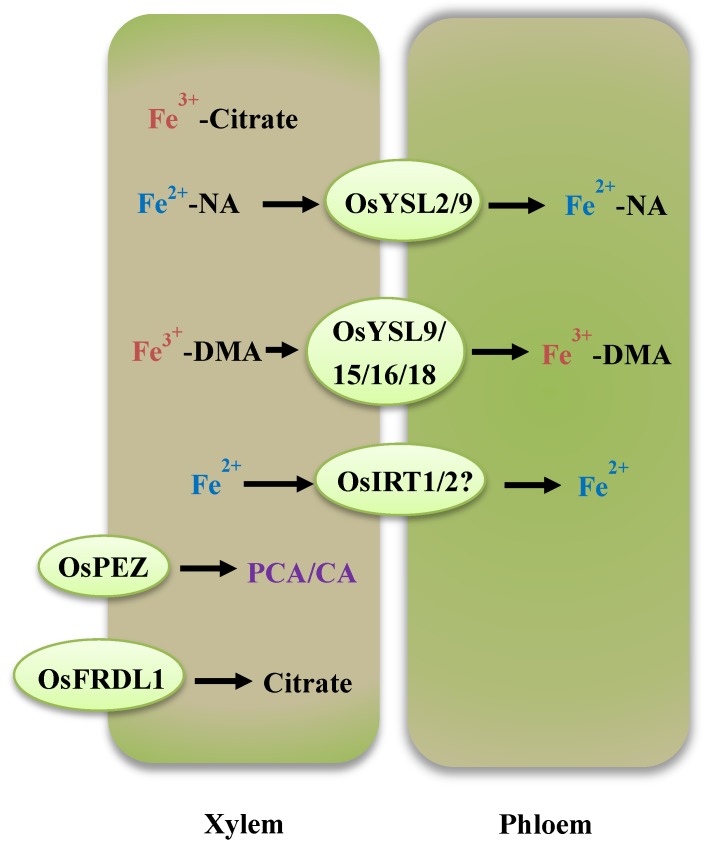
A simplified model of internal Fe translocation in rice. Citrate, NA, and DMA are the main chelators used to bind Fe within rice. OsFRDL1 encodes a citrate effluxer. OsYSL transporters are responsible for the translocation of Fe^3+^-DMA and Fe^2+^-NA from xylem to phloem. Xylem and phloem are shown in rounded rectangles. Transporters are shown in light green ellipses.

Once absorbed at the root’s surface, Fe is radially transported across the root epidermis, cortex, and pericycle to reach the xylem parenchyma cell, where Fe is unloaded into the xylem vessels by Fe transporters [8,65]. Due to its low solubility and toxicity to cells, Fe needs to be complexed with a suitable chelator within the plant body. Citrate [66,67], NA [68], and MAs [37] are demonstrated to be the main chelators that bind Fe in plants. Citrate plays a dominant role in the chelating and trafficking of Fe in xylem sap in some plants, such as soybean, tomato, rice, and *Arabidopsis* [66,69]. In recent years, the transporters of citrate have been identified. By analyzing the *Arabidopsis* mutant, *frd3*, AtFRD3, a member of the multidrug and toxic compound extrusion (MATE) family, was shown to play an important role in xylem Fe transport. Compared with wild-type plants grown on Fe-sufficient media, an *frd3* mutant grown under identical conditions exhibits chlorotic leaves and a significant reduction of citrate and Fe in the xylem exudate. Furthermore, the phenotypes of *frd3* mutants can be rescued when grown on citrate-supplemented media. These results suggest that AtFRD3 is involved in loading citrate in the xylem. *OsFRDL1*, an *AtFRD3*-like gene in rice, encodes a citrate effluxer that is specifically expressed in root pericycle cells [38,70]. The expression of *OsFRDL1* shows no obvious responses to Fe deficiency treatment. *Osfrdl1* mutants exhibit Fe deficiency-induced chlorosis due to lower Fe concentrations in their leaves, and the amounts of citrate and ferric iron in the xylem sap of *osfrdl* mutants was also less than the amounts in the wild-type rice [70]. When grown under Fe-deficient conditions, *osfrdl* mutants contain much greater Fe concentrations in their roots compared to wild-type rice [70]. Using the Perls blue staining method, Fe^3+^ was observed to deposit in the root stele of *osfrdl* mutants [70]. At the stage of reproduction, Yokosho et al. reported that *OsFRDL1* shows significant expression in the upper nodes of rice plants. The pollen viability and grain yield of the *Osfrdl1* mutant was reduced, which may be accounted for by the enhanced Fe precipitation in node I and the impaired distribution of Fe to the panicles [71]. These results suggest that *OsFRDL1* functions during the translocation of Fe from roots to shoots in the period of vegetative growth, as well as during the distribution of Fe to panicles at the period of reproduction (Figure 2). To solubilize the deposited apoplasmic Fe, various phenolics, including PCA and CA, are extruded in plants [39,40]. In rice, OsPEZ1 and OsPEZ2 are identified as two phenolic efflux transporters (Figure 2). These two transporters can facilitate loading phenolics into the xylem, thus prompting the utilization of deposited apoplasmic Fe from root surfaces into the stele. Overexpression of *OsPEZ1* leads to Fe toxicity phenotypes, as evidenced by severely reduced growth and leaf-tip necrosis, which are attributed to the increased contents of Fe in plants body under Fe-replete conditions [39,40]. *ospez1/2* mutants have higher amounts of apoplasmic Fe in roots, thereby exhibiting a greater tolerance to Fe-deficient conditions than wild-type rice plants [39,40].

After Fe is loaded into xylem sap, a part of the Fe will be unloaded into the phloem cell, through which Fe is transported to various plant tissue [67]. An iron transporter is necessary for this process. In recent years, the YSL family has been reported to not only facilitate Fe^3+^-DMA uptake from the rhizosphere but is also involved in iron unloading into the phloem [60]. OsYSL2 is a member of the YSL family and is required to transport Fe^2+^-NA and Mn^2+^-NA but not Fe^3+^-DMA. The expression of OsYSL2 is detected in phloem cells and developing seeds [31]. By analyzing transgenic plants with altered expression levels, OsYSL2 has been shown to affect the distribution of Fe within the plants [31,32]. Collectively, these results suggest that OsYSL2 is a pivotal Fe^2+^-NA transporter mediating the phloem transport of Fe in rice [31,32]. OsYSL9 also belongs to the YSL transporter family and is responsible for the transport of both Fe^2+^-NA and Fe^3+^-DMA across the plasma membrane. Treatment with Fe deficiency increases the expression of *OsYSL9* in the central cylinder instead of the epidermis. *OsYSL9* RNAi transgenic rice is less tolerant to Fe deficiency than wild-type rice plants, as evidenced by their shorter shoot lengths and the lower concentrations of chlorophyll and Fe in their leaves. In particular, knocking down the expression of *OsYSL9* reduces and improves the concentration of Fe in embryos and brown seeds without embryos. These results suggest that *OsYSL9* participates in iron translocation within the rice plant body, especially in developing seeds [33]. Recently, another YSL transporter, *OsYSL13*, was reported to regulate the translocation of Fe to younger leaves and seeds in rice [34]. OsYSL15 is a Fe^3+^-DMA transporter and play a critical role in Fe uptake from the rhizosphere. The expression of *OsYSL15* is detected in vascular bundles and developing seeds, implying that OsYSL15 may be responsible for the translocation of Fe in the phloem and loading of seeds [35,72]. *OsYSL16* is highly similar to *OsYSL2* and *OsYSL15* and has the ability to acquire Fe^3+^-DMA from the rhizosphere. In addition, *OsYSL16* is highly expressed in vascular bundles and mediates the internal distribution of Fe^3+^-DMA in rice [36,73]. The *osysl16* mutants tend to deposit Fe mainly in the veins and are less tolerant to Fe deficiency than wild-type plants [36,73]. *OsYSL18* is also localized in the plasma membrane with the function of translocating Fe^3+^-DMA. OsYSL18 is reported to take part in the transport of Fe inside the reproductive organs and joins the phloem of rice [37]. Like *OsYSL15*, the expression of *TOM1* and *OsIRT1* can be detected in the vascular tissues of rice plants, which indicates that these two transporters function in the Fe acquisition from the rhizosphere, as well as internal Fe distribution [18,29] (Figure 2).

## 4. Regulation of Fe Deficiency Responsive Genes

**Figure 3:**
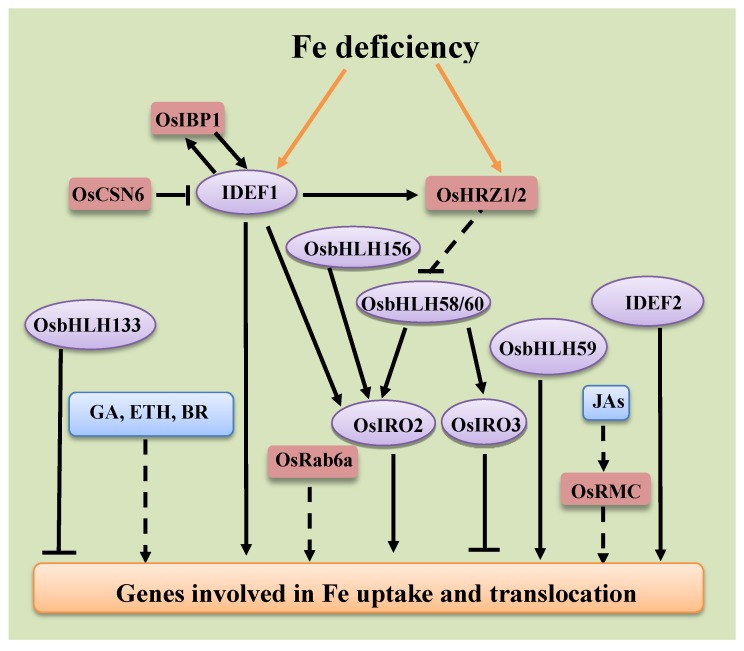
The regulatory networks of the genes involved in Fe uptake and translocation in rice roots. Transcription factors are shown in light purple ellipses. Other regulatory proteins are shown in pink rounded rectangles. Hormones and signaling molecules are shown in light blue rounded rectangles. The genes involved in Fe uptake and translocation are shown in light orange rounded rectangles. Positive regulation is indicated by black arrows. Negative regulation is indicated by black blunt arrows. Broken lines indicate regulation with unknown mechanisms. Orange lines indicate that Fe signals are sensed by IDEF1, OsHRZ1, and OsHRZ2.

To maintain Fe homeostasis, plants develop a signaling network to regulate iron absorption and transport. There are two cis-acting elements, IDE1 and IDE2, which exist at the promoters of Fe deficiency responsive genes in a number of plant species. In rice, two transcription factors, IDEF1 and IDEF2, have been identified to specifically bind IDE1 and IDE2, respectively [41,43] (Figure 3). IDEF1 belongs to the ABI3/VP1 (abscisic acid insensitive 3/viviparous 1) transcription factor, while IDEF2 is a member of the transcription factor NAC family [41,43]. The constitutive expression of *IDEF1* and *IDEF2* is detected in vegetative and reproductive tissues [42,43,74]. The genes responsible for Fe uptake and translocation are regulated by IDEF1 under Fe-sufficient conditions, as well as in the early stages of Fe deficiency. At subsequent stages of Fe deficiency, IDEF1 can affect the expression of LEA (late embryogenesis abundant) genes by binding the RY ***cis*** element (CATGCA) [42]. In addition, IDEF1 is thought to be an Fe sensor due to its ability to directly bind Fe via the metal-binding domain, which is essential for the Fe deficiency signaling cascade [75]. In contrast to IDEF1, the target genes of IDEF2 are not altered during the processes of Fe-deficiency [74]. IDEF2 is required for the expression of a number of Fe deficiency-induced genes. In addition, *OsYSL2* has been shown to be the target gene of IDEF2, which recognizes IDE2 sties in the promoter of *OsYSL2*. The *IDEF2* knockdown transgenic rice exhibits abnormal Fe allocation between the shoots and roots [74].

OsHRZ1 and OsHRZ2 are also regraded as candidates for Fe sensors because they directly bind Fe via hemerythrin domains. OsHRZ1 and OsHRZ2 also have RING (really interesting new gene) Zn-finger domains that act as E3 ubiquitin ligases [51,76]. *OsHRZ1* and *OsHRZ2* RNAi rice plants show enhanced tolerance to Fe deficiency treatment compared to wild-type rice plants, coupled with the increased contents of Fe in shoots and grains, as well as Fe deficiency responsive genes [51,76]. Thus, OsHRZ1 and OsHRZ2 are assumed to negatively modulate the responses of rice to Fe deficiency (Figure 3). Additionally, the expression of both *OsHRZ1* and *OsHRZ2* is under the control of IDEF1 in rice. OsHORZ1 is also a protein that contains the hemerythrin domain and is suggested to repress OsHRZ functions [51,76].

OsIBP1.1 and OsIBP1.2 are Bowman–Birk trypsin inhibitors, which can interact with IDEF1 and thus prevent the 26S proteasome-mediated degradation of IDEF1 (Figure 3). Fe-deficient treatments induce the expression of *OsIBP1.1* and *OsIBP1.2* via IDEF1. An overexpression of *OsIBP1.1* markedly increases the expression of *OsYSL2* in rice [52]. The OsCOP9 complex consists of eight subunits involved in the ubiquitin–proteasome degradation processes. Among these eight subunits, OsCSN6 is repressed at an early stage of Fe deficiency in rice, which decreases the activity of the OsCOP9 complex. Knockdown of OsCSN6 leads to the accumulation of IDEF1, which subsequently enhances Fe-related genes at early stages of Fe deficiency in rice [55].

The bHLH (basic helix-loop-helix) transcription factor family plays a critical role in the regulation of Fe-deficiency response genes in both strategy I and strategy II plants [60]. In rice, there are several bHLH transcription factors that have been characterized to be regulators of Fe homeostasis (Figure 3). Among them, *OsIRO2* is a well-studied bHLH transcription factor in rice [44]. The overexpression of *OsIRO2* in rice increases MA secretion and Fe content in shoots, and thus improves the tolerance of rice plants to Fe deficiency [77,78]. A microarray analysis shows that the interaction between OsIRO2 and IDEF1 positively modulates many Fe deficiency responsive genes, and its expression may be regulated by IDEF1 [41,42]. OsIRO3 is also a bHLH transcription factor and is identified as a negative regulator of Fe utilization genes. *OsIRO3*-overexpression rice plants are very sensitive to Fe deficiency treatment because of their reduction in the expression of genes involved in Fe uptake and translocation [45]. Another bHLH transcription factor, OsbHLH133, has been demonstrated to play a negative regulatory role in the distribution of Fe between the shoots and roots of rice plants. Specifically, the *osbhlh133* mutant exhibits decreased Fe concentrations in its shoots and increased Fe concentration in its roots due to its higher expression of the genes involved in Fe uptake and translocation than wild-type plants [49]. However, the Fe concentration in the shoots and xylem sap of *OsbHLH133*-overexpressing rice plants is suppressed while the Fe concentration in roots is improved [49]. OsbHLH156 is found to positively regulate Fe deficiency response genes by facilitating nuclear localization of OsIRO2 [50]. There are four members of the IVc bHLHs subgroup in rice, *OsbHLH57*, *OsbHLH58, OsbHLH59,* and *OsbHLH60* [46,47,48]. Zhang et al. reported that *OsbHLH60* (also named *OsPRI1*) positively modulates the responses of rice to Fe deficiency [48]. Compared to wild-type plants, *osphr1* mutants exhibit less tolerance to Fe deficiency due to their inhibited expression of Fe deficiency inducible genes. OsPRI1 is found to interact with, and suggested to be ubiquitinated by, OsHRZ1. The knockout of *OsPRI1* can decrease the tolerance of *oshrz1* mutants to Fe deficiency. Furthermore, OsPRI1 acts upstream of OsIRO2 and OsIRO3. Taken together, a signaling pathway underlying the regulation of Fe deficiency responses is proposed as OsHRZ1-OsPRI1-OsIRO2/3 [48]. Kobayashi et al. found that OsbHLH58 and OsbHLH59 are two positive regulators of Fe responses, such that the knockout of these two transcription factors decreases the expression of Fe deficiency response genes and impairs the tolerance of rice plants to Fe deficiency treatments [46]. They uncovered strong interactions between OsbHLH58 and OsHRZs (OsHRZ1 and OsHRZ2) and no interaction between OsbHLH59 and OsHRZs. OsbHLH58, OsbHLH59, and OsbHLH60 were not found to be ubiquitinated by OsHRZs [46]. *OsbHLH57* is also induced by Fe deficiency in the shoots and roots of rice plants [46]. A detailed study of *OsbHLH057* in rice responses to Fe deficiency is warranted. Recently, Zhang et al. reported partially inconsistent results with those of Kobayashi et al. They found that OsHRZ1 directly interacts with OsbHLH058 and OsbHLH059 and promotes their degradation [47]. These inconsistent results may be due to the different experimental methods used in these studies.

OsRab6a and OsRMC are also regulators of Fe-deficient responses (Figure 3). OsRab6a is a small GTPase in rice [53]. Under Fe-deficient conditions, *OsRab6a* overexpression lines have higher Fe concentrations in the shoots and roots of grains by up-regulating *OsIRO2*, *OsIRT1*, *OsNAS1*, and *OsNAS2*, suggesting that OsRab6a plays an important role in the regulation of Fe acquisition in rice plants [53]. OsRMC is a receptor-like protein, which was previously found to be involved in JA (jasmonic acid)-mediated root development. Through comparison with wild-type and transgenic plants under Fe-deficient conditions, OsRMC is shown to be a positive regulator of Fe uptake in rice [54]. IMA (IRON MAN, a short C-terminal amino-acid sequence consensus motif) genes encode Fe-responsive peptides in the phloem. There are two IMA genes in rice, *OsIMA1* and *OsIMA2*. The heterologous expression of *OsIMA1* in arabidopsis increases the Fe concentration in rosettes by promoting FCR (ferric-chelate reduction) activity under Fe-sufficient conditions [56].

## 5. Regulatory Roles of Phytohormones and Messenger Molecules in Fe Homeostasis

Various phytohormones and messenger molecules have been demonstrated to regulate the Fe deficiency responses in rice. The following section presents a compilation of emerging information on the functions of phytohormones and messenger molecules in responding to Fe deficiency in rice (Figure 3).

### 5.1. Brassinosteroids (BRs)

Brassinosteroids (BRs) are a type of steroid hormone that plays an important role in plant growth and development [79]. Emerging studies shed light on the regulatory roles of BR in maintaining Fe homeostasis in plants. Wang et al. reported that BRs participate in the regulation of Fe deficiency responses in cucumber (*Cucumis sativus*). They found that BRs may negatively modulate Fe homeostasis by reducing Fe deficiency-induced FRO activities, the expression level of two Fe deficiency responsive genes (*CsFRO1* and *CsIRT1*), and the Fe translocation from root to shoot in cucumbers (*Cucumis sativus*) [80]. Then, these researchers found that BRs are also involved in the responses of rice to Fe deficiency at the physiological and molecular level. Specifically, the exogenous application of EBR (24-epibrassinolide, a brassinosteroid) can produce more evident chlorosis in rice leaves and reduce the growth of seedlings under Fe deficiency conditions. In addition, EBR changes the translocation of Fe in the phloem, leading to a greater accumulation of Fe in the roots under both Fe-sufficient and Fe-deficient conditions. These responses of rice treated with EBR under Fe deficiency conditions can be accounted for by the expression patterns of Fe-deficient responsive genes, such that, in the roots, *OsIRT1*, *OsYSL15*, *OsYSL2*, *OsNAS1,* and *OsNAS2* are induced by EBR, while in shoots, the genes *OsYSL2*, *OsNAS1,* and *OsNAS2* are suppressed by EBR under Fe-deficient conditions [81].

### 5.2. Gibberellins (GAs)

Gibberellins (GAs) are a classical type of phytohormone that functions in the regulation of various aspects of physiological processes [82]. In arabidopsis, GA signaling is found to be involved in Fe deficiency responses by positively modulating the expression of the Fe-uptake gene [83]. However, there is limited information available on the involvement of GAs in the regulation of Fe homeostasis in rice. The protein OsEUI (elongated uppermost internode) is a GA-deactivating enzyme, and a mutation of this gene leads to an increase in the GA concentration in rice. By comparing wild-type with *eui* mutant and EUI overexpression lines, GA is demonstrated to be involved in the responses of rice to Fe deficiency by negatively modulating Fe transport and translocation from root to shoots. Treatment with GA could exaggerate leaf chlorosis and suppress growth, which is attributed to the reduction of Fe concentration in leaves under Fe deficiency conditions. In wild-type roots, some Fe deficiency responsive genes including *OsIRO2, OsNAAT1* and *OsYSL15* are induced by GA treatment under Fe deficiency. The concentration of Fe in the shoots of the EUI overexpression line and *oseui* mutant is higher and lower than that of the wild-type plants, respectively. In addition, the retardation of Fe translocation in the shoots of the *oseui* mutant may be due to a decrease in the *OsYSL2* expression level [84].

### 5.3. Jasmonates (JAs)

Jasmonates (JAs), includingJA, and their biologically active derivatives, are synthesized from α-linolenic acid in plants when exposed to biotic and abiotic stresses [85,86]. In arabidopsis, JA was discovered to suppress IRT1 and FRO2 expression when exposed to Fe deficiency [87]. Some studies have provided new insights into the involvement of JAs in the responses of rice to Fe deficiency. *OsRMC* encodes a receptor-like protein and can alter root development by targeting the JA signaling pathway. Our study demonstrates that the overexpression of *OsRMC* enhances Fe concentration and upregulates the expression level of Fe deficiency-related genes, including *OsDMAS1*, *OsNAS1*, *OsNAS2*, *OsNAAT1*, *OsIRT1*, *OsYSL15,* and *OsIRO2* in rice seedlings [54]. OsIBP1.1 and OsIBP1.2 are Bowman–Birk trypsin inhibitors and can bind the transcription factor IDEF1. The expressions of these two homologous genes are induced by Fe deficiency, as well as by the exogenous application of JA [52,88]. Kobayashi et al. investigated the regulatory roles of JA in the responses to Fe deficiency in rice roots. They found that very early Fe deficiency could rapidly increase the concentration of endogenous JAs and activate the JA signaling pathways via OsHRZs and IDEF1 in rice roots. *cpm2* is a rice JA-deficient mutant. By comparing *cpm2* and the wild-type, JA was shown to down-regulate Fe deficiency responsive genes under Fe-sufficient conditions, but this downregulation could be ameliorated under Fe deficiency conditions [89]. Taken together, these results suggest that JA plays a positive role in the regulation of Fe uptake and translocation during early periods of Fe deficiency treatment in rice roots [89].

### 5.4. Ethylene

Ethylene is a gaseous hormone that plays very important roles in plant growth and development [90]. Recently, ethylene was reported to be involved in the regulation of Fe deficiency responses in rice [91,92]. When exposed to Fe-starvation conditions, the expression levels of several ethylene biosynthesis-related genes encoding ACS (1-aminocyclopropane-1-carboxylic acid (ACC) synthases) and ACO (ACC oxidases), as well as the production of ethylene, are significantly enhanced in the roots of rice plants [92,93]. The exogenous application of the ethylene precursor ACC (1-aminocyclopropane-1-carboxylic acid) could increase the concentration of soluble Fe in rice seedlings to alleviate Fe deficiency-induced leaf chlorosis. The genes involved in Fe acquisition are significantly induced by ethylene under Fe-depleted conditions but are repressed by ethylene inhibitors, such as Co^2+^ and STS (silver thiosulphate). An analysis of *OsIRO2* RNAi transgenic rice reveals that the upregulation of *OsNAS1*, *OsNAS2*, *OsYSL15,* and *OsIRT1* by ethylene treatment is dependent on *OsIRO2* [92].

### 5.5. Sucrose

As the main product of plant photosynthesis, sucrose acts as an energy resource for growth and development, as well as a signaling substance that participates in the responses of plants to biotic and abiotic stresses, such as mineral starvation [94,95]. In rice, sucrose is found to take part in modulating Fe deficiency responses [94]. A greater amount of sucrose tends to accumulate in the leaves rather than in the roots of rice plants when grown under Fe-deficient conditions by suppressing the transcript abundance of *SUT* (sucrose transporter) genes in leaves. Additionally, the exogenous application of sucrose and a reduction in endogenous sucrose concentrations by dark treatment led to opposite expression patterns for Fe-related genes in roots and leaves under Fe deficiency conditions. These results highlight that sucrose functions as a shoot-generated signaling substance mediating shoot–root communication in the responses of rice plants to Fe deficiency [94].

### 5.6. Auxin

Auxin has also been proven to participate in the responses of rice to Fe deficiency. Liu et al. found that treatment with auxin exaggerates the Fe deficiency characteristics in rice [96]. This auxin signaling is responsible for the suppression of growth and the photosynthesis of rice plants when subjected to Fe-deficient treatments [96]. In addition, some auxin response-related genes have been shown to participate in the crosstalk between auxin signaling and Fe deficiency responses. OsABCB14, an ATP Binding Cassette B/Multidrug-Resistance/P-glyco-protein (ABCB/MDR/PGP) regulating auxin transport, affects Fe acquisition in rice [97]. ARF (auxin response factor) belongs to multiple gene families and mediates the actions of auxins. Based on an analysis of the *osarf12* mutant, the transcription factor OsARF12 could alter Fe accumulation via regulation of the *OsIRT1* in rice [98]. Transcription factor OsARF16 is another member of the ARF family, which has been reported to adjust auxin redistribution and Fe deficiency responses in rice [99].

### 5.7. Nitric Oxide (NO)

Nitric oxide (NO) plays a critical role in the regulation of Fe deficiency responses in plants [100]. However, less attention is paid to the function of NO in the Fe uptake of strategy II plants compared to the great number of studies on strategy I plants [100]. Recently, Sun et al. observed that NO is produced by the NO synthase-like pathway and acts downstream of auxin to modify root growth under Fe deficiency conditions [101]. Moreover, Zhu et al. reported that NO mediates cell wall Fe reutilization when rice plants grow under Fe deficiency conditions in the presence of NH_4_^+^ [102].

### 5.8. Abscisic Acid (ABA)

Abscisic acid (ABA) is a well-documented plant hormone that plays important roles in responses to multifaceted stresses [103]. In Arabidopsis, ABA was shown to alleviate leaf chlorosis symptoms via the reutilization of root Fe and the translocation of Fe from root to shoot under Fe deficiency. In contrast, only limited clues indicate the role of ABA in response to Fe deficiency in rice [41,42]. As mentioned above, IDEF1 is a member of the ABI3/VP1 transcription factor family participating in the ABA response in plants [41]. In addition, several ABA-responsive genes are induced by Fe deficiency through IDEF1 in rice [42].

### 5.9. Cytokinins (CKs) and Salicylic Acid (SA)

Cytokinins (CKs) play a negative role in the modulation of the iron acquisition process in the root of Arabidopsis [104]. Salicylic acid (SA) is another phytohormone that is also reported to be involved in the regulation of Fe homeostasis in Arabidopsis [105]. However, to the best of our knowledge, no research has been conducted to investigate the roles of CKs and SA in the responses of rice to Fe-deficient conditions.

## 6. Conclusions

Rice is highly susceptible to Fe deficiency in soil and accumulates relatively low amounts of Fe in polished seeds, which threatens human health. In this review, the molecular mechanisms modulating Fe homeostasis in rice were summarized. Over the past years, great progress has been made to identify the genes responsible for Fe uptake, translocation, and reutilization in rice. In addition, phytohormone and signaling molecules have also been demonstrated to play important roles in the regulation of Fe deficiency response genes in rice. Despite this progress, more efforts are still needed to understand the mechanisms underlying the responses of rice to Fe deficiency at the transcriptional and post-transcriptional levels. These efforts will subsequently facilitate Fe biofortification in rice grains via molecular breeding methods

## Figures and Tables

**Table 1:** Genes involved in iron (Fe) uptake and transport in rice.

Gene Name	Gene ID	Function	References
DMA biosynthesis			
*OsNAS1*	Os03g0307300	Nicotianamine synthase	[26]
*OsNAS2*	Os03g0307200	Nicotianamine synthase	[26]
*OsNAS3*	Os07g0689600	Nicotianamine synthase	[26]
*OsNAAT1*	Os02g0306400	Nicotianamine aminotransferase	[27]
*OsDMAS1*	Os03g0237100	Deoxymugineic acid synthase	[28]
Transporters			
*OsTOM1*	Os11g0134900	DMA efflux transporter	[29]
*OsTOM2*	Os11g0135000	DMA efflux transporter	[30]
*OsYSL2*	Os02g0649900	Fe^2+^-NA transporter	[31,32]
*OsYSL9*	Os04g0542200	Fe^2+^-NA and Fe^3+^-DMA transporter	[33]
*OsYSL13*	Os04g0524500	Involved in Fe distribution	[34]
*OsYSL15*	Os02g0650300	Fe^3+^-DMA transporter	[35]
*OsYSL16*	Os04g0542800	Fe^3+^-DMA transporter	[36]
*OsYSL18*	Os01g0829900	Fe^3+^-DMA transporter	[37]
*OsFRDL1*	Os03g0216700	Citrate efflux transporter	[38]
*OsIRT1*	Os03g0667500	Fe^2+^ transporter	[18]
*OsIRT2*	Os03g0667300	Fe^2+^ transporter	[18]
*PEZ1*	Os03g0571900	Phenolics efflux transporter	[39]
*PEZ2*	Os03g0572900	Phenolics efflux transporter	[40]
Transcription factors			
*IDEF1*	Os08g0101000	Positive transcriptional regulator	[41,42]
*IDEF2*	Os05g0426200	Positive transcriptional regulator	[43]
*OsIRO2*	Os01g0952800	Positive transcriptional regulator	[44]
*OsIRO3*	Os03g0379300	Negative transcriptional regulator	[45]
*OsbHLH58*	Os05g0455400,	Positive transcriptional regulator	[46,47]
*OsbHLH59*	Os02g0116600,	Positive transcriptional regulator	[46,47]
*OsbHLH60*	Os08g0138500	Positive transcriptional regulator	[48]
*OsbHLH133*	Os12g0508500	Negative transcriptional regulator	[49]
*OsbHLH156*	Os04g0381700	Positive transcriptional regulator	[50]
Other genes			
*OsFRO1*	Os04g0444800	Fe^3+^-chelate reductase	[18]
*OsHRZ1*	Os01g0689300	Ubiquitin ligase	[51]
*OsHRZ2*	Os05g0551000	Ubiquitin ligase	[51]
*OsHORZ1*	Os01g0861700	Haemerythrin domain containing protein	[51]
*OsIBP1.1*	Os01g0124200	Bowman–Birk Trypsin Inhibitor	[52]
*OsIBP1.2*	Os01g0124400	Bowman–Birk Trypsin Inhibitor	[52]
*OsRab6a*	Os03g0191400	Small GTPase	[53]
*OsRMC*	Os04g0659300	Receptor-like protein	[54]
*OsCSN6*	Os08g0500000	COP9 signalosome subunit 6	[55]
*OsIMA1*	Os01g0647200	Fe-responsive peptides	[56]
